# Slit in a Nest Site Influences the Nest Site Selection in Cavity Nesting Ant Colonies

**DOI:** 10.3390/insects15090638

**Published:** 2024-08-26

**Authors:** Anna Gruszka, Mateusz Rolski, Mariia Marczak, Sławomir Mitrus

**Affiliations:** Institute of Biology, University of Opole, Oleska 22, 45-052 Opole, Poland; ania4504@gmail.com (A.G.); matirol@op.pl (M.R.); marczakmam@gmail.com (M.M.)

**Keywords:** *Temnothorax crassispinus*, hymenoptera, formicidae, cavity nesting ants, acorn ants, nest choice

## Abstract

**Simple Summary:**

Ants are a widespread and highly abundant group of animals in almost all terrestrial ecosystems. Nesting sites are important for ants, as the nest protects them against predators and can ensure optimal conditions for the brood development. We studied the nest site selection by *Temnothorax crassispinus* ant colonies, which typically inhabit empty acorns. During this study, we used artificial nest sites with slits in the nest wall, mimicking the cracks in acorns under natural conditions. We found that the ant colonies preferred artificial nest sites without these slits. The absence of cracks in acorns could be an indication of the durability of potential nest sites; thus, we propose that choosing acorns without such damage could be beneficial for the ant colonies.

**Abstract:**

For ants, nests provide a refuge against predators and protection from environmental factors. Thus, choosing a good nest site is important for an ant colony, but nest sites are limited resources. Ants of the genus *Temnothorax* inhabit small cavities in, e.g., acorns, twigs and under rocks. Earlier, it was shown that the ants are able to choose a superior site. In this study, using binary choice tests, we studied the nest site selection by *Temnothorax crassispinus* ant colonies that typically inhabit empty acorns. For this purpose, we used artificial nest sites without and with an additional slit in the nest wall, mimicking the cracks in potential nest sites under natural conditions. We found that the ant colonies preferred artificial nest sites without these slits. However, no difference in the number of colonies inhabited nest sites with a slit vs. those without a slit was found when the slits were closed using transparent food foil, which prevented the air flow while keeping an inflow of light. What is more, additional light through the hole in the red filter covering the artificial nest sites had no influence on the nest site selection. The results of this study suggest that the air flow through a slit in the nest site wall, rather than additional light, influences the nest site selection. The absence of cracks, e.g., in acorns, could be an indication of the durability of potential nest sites. Thus, choosing a cavity without such damage could be beneficial for the ant colonies.

## 1. Introduction

Ants (Hymenoptera: Formicidae) are a widespread group of animals, with presently over 14,000 known species [1]. They are typically highly abundant in almost all terrestrial ecosystems. Ants are typically general predators, but they are also a significant food source for many animals, so they have an impact on seed dispersal, pollination and the processes of soil formation. Thus, ants have considerable impact on ecosystems (e.g., [2,3,4]). 

An important part of ants’ ecology is their nesting sites. For ants, nests provide a refuge against predators, as well as protection from environmental factors such as extreme temperatures and desiccation, and they can ensure optimal conditions for the brood development while also influencing reproduction and competition with other colonies [2,5]. Thus, many ant species construct nests, which can be large structures with numerous chambers and entrances, such as the anthills of the mound-building *Formica* species groups [2,6,7]. However, other species—known as cavity nesting ants—do not construct nests but inhabit available spaces, including cavities in wood, seeds or under rocks. For these ants, finding a good nest site is crucial, but good-quality cavities are usually limited resources [5,8].

Examples of cavity nesting ants are species of the genus *Temnothorax*. The ants of this genus are small—the length of a worker is about 2–3.5 mm—and they live in small colonies, typically reaching a few dozen to two hundred individuals [6,9]. Because of the colony size and well-established protocols for rearing them in the laboratory (cf. [10]), these ants are frequently used to study ant ecology; for example, these ants have been used in numerous laboratory experiments on nest site selection using artificial nest sites. It was shown that the ant colonies prefer sites with a narrow entrance, which could be easier to defend, and generally, they prefer larger cavities, tall versus flat sites and dark versus light ones (for example: [11,12,13,14]). It was also shown that *Temnothorax* ants are able to choose a superior site, even if it is located at a greater distance than a nearby nest site of a lower quality [15], and they can choose a cavity on the basis of multiple features [16]. 

Under natural conditions, differences between potential nest sites can be weaker than those between the artificial nest sites used during some laboratory experiments—such as for the example mentioned above difference between dark vs. light sites. What is more, if necessary, cavity nesting ants can modify the sites that are already available. For example, the ants can inhabit cavities in empty acorns and twigs, which usually have a hole resulting from the activity of a wood-boring beetle [17,18,19]. The hole is used by the ants as an entrance, but if such a hole is too large, it could be modified by the ants—such behavior was observed in numerous species of cavity nesting ants, including *Temnothorax* ants (e.g., [11,20,21]). However, empty old acorns—frequently used by the ants as nesting sites—could also have cracks [22]. Such cracks are probably more difficult to repair than just reducing the size of the nest entrance; additionally, such cracks could have a strong effect on the durability of the acorns. Thus, distinguishing between potential nest sites with and without cracks could be favorable for the ant colonies. It was proposed that ants favor a dark interior, because it is a cue of the nest wall’s integrity [14]—so darker nest sites could be perceived as sturdier and thus better for the ant colonies. However, recognizing nest sites with cracks could be based on visual signals but also the flow of air or using the sense of touch. In this study, the following questions were posed: (1) Are the cavity nesting ants able to distinguish between nest sites ‘with a slit’ and those without ‘with a slit’?; and (2) If so, is such a preference based on light or rather the air flow through the ‘slit’? For this study, we performed laboratory binary choice tests.

## 2. Materials and Methods

During this study, colonies of the acorn ant *Temnothorax crassispinus* were used. This ant species is present throughout central and eastern Europe, and it is widely distributed throughout Poland. It lives in light coniferous and mixed forests [6,23]. Colonies of the ant are small, typically numbering from a few dozen to about 200 workers. They mostly inhabit cavities in old acorns and fallen sticks [6,23,24]. 

For this study, we collected acorns containing ant colonies near Opole, Poland (GPS: 50.624698, 18.108744). In the field, we put each collected acorn into a separate plastic box and transported it to a laboratory. In the laboratory, we opened the acorns and then captured the ants with an aspirator and counted them. The colonies chosen for the experiments (see below) were transferred to square Petri dishes (10.2 cm × 10.2 cm × 1.9 cm). Each dish had a thin plaster base with two artificial nest sites placed on top. After each experiment, the base was removed and the dishes were carefully cleaned with dishwashing liquid. 

**Figure 1:**
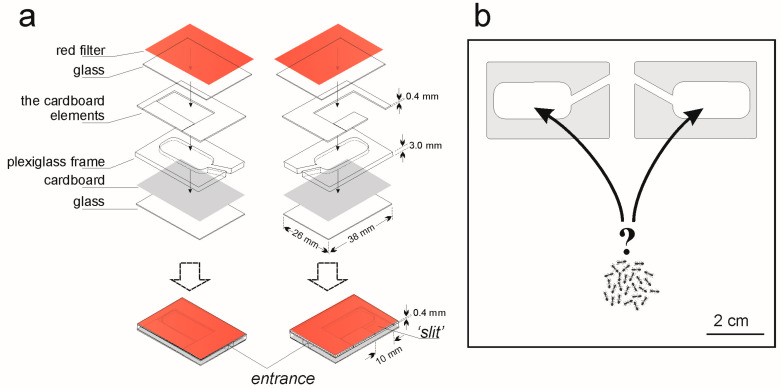
Nest design used in the study and a scheme of binary choice tests using the cavity nesting ant *Temnothorax crassispinus* colonies. (**a**) Nest design: a cavity between a piece of cardboard and half of a microscope slide (38 mm × 26 mm), separated by a plexiglass frame (3 mm thick) and cardboard elements (0.4 mm thick; see Figure 2a), and covered with a piece of red translucent filter; (**b**) scheme of the binary choice tests: in square Petri dishes (10.2 cm × 10.2 cm × 1.9 cm) two artificial nest sites were placed; the ant colonies were placed at a similar distance (approximately 7 cm) from the entrances to the two artificial nest sites.

The artificial nest sites used for this study comprised a cavity between a piece of cardboard and half a microscope slide, separated by a plexiglass frame (3 mm thick) and cardboard elements (0.4 mm thick), and covered with a piece of red translucent filter (Figure 1a); the height of 0.4 mm (i.e., the thick cardboard elements above the plexiglass frame) was too small for the ants to enter through there. After the experiment, the microscope slides and plexiglass frames used to prepare nest sites were carefully cleaned with an alcohol and dishwashing liquid; the pieces of cardboard were used only once. 

During the preference tests, the distances between the entries to the nest were approximately 1 cm (see Figure 1b). We performed three binary choice experiments; for each, we used 36 ant colonies. Any ant colony was used in one experiment only.

Experiment 1. If a ‘slit’ in the nest side influences the nest site selection

**Figure 2:**
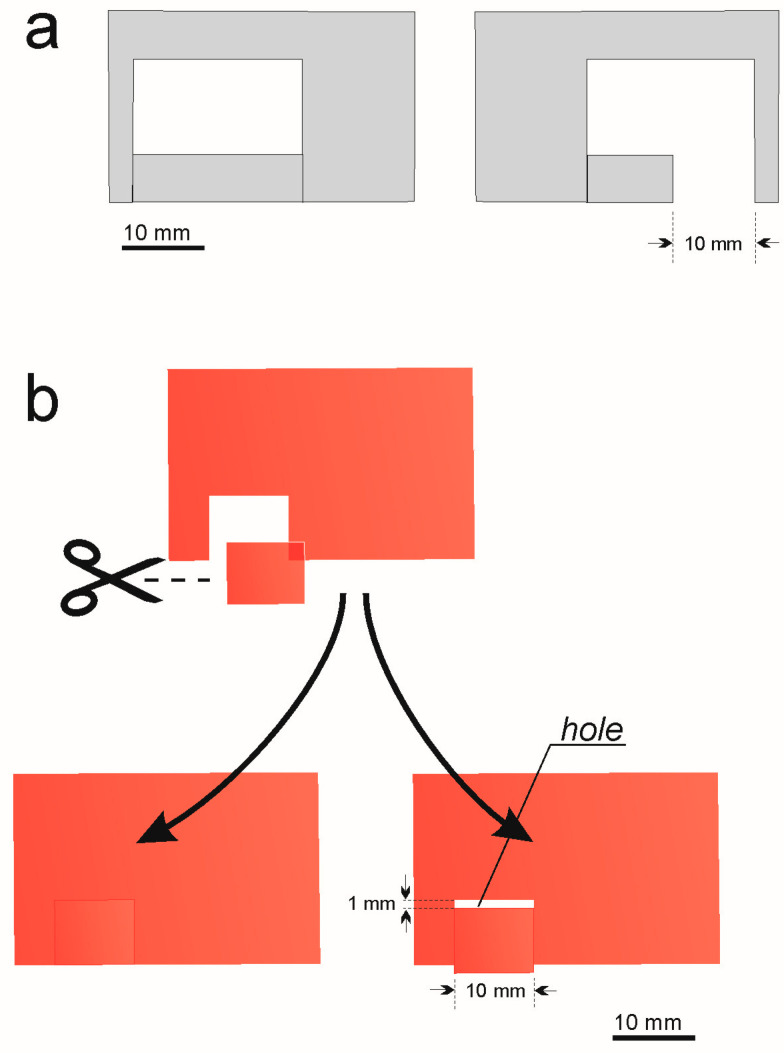
Scheme of the elements used during the study: (**a**) cardboard elements (thickness 0.4 mm) used for the construction of the artificial nest sites (see Figure 1a); (**b**) scheme of the pieces of red translucent filter—the pieces were used to cover the nest sites (see Figure 1a) during one of the binary choice tests (see the text for details).

On 6 June 2023, we collected 48 acorns with ant colonies (i.e., ants with a brood in different stages of development): 22 queenless; 25 containing one queen; and one with two queens (workers: 6–116, median: 39.5). For the experiment, we chose the largest colonies, i.e., those containing minimum 20 workers, median: 45 (14 queenless colonies; 21 containing one queen; and one with two queens). During the experiment, two artificial nest sites were placed in each Petri dish: one of these sites had a ‘slit’ in a wall, while the second one was without such a ‘slit’ (see Figure 1a and Figure 2a).

Experiment 2. If a ‘slit’ covered by food foil influences the nest site selection

On 20 June 2023, we collected 61 acorns with ant colonies: 27 queenless and 34 queenright (workers: 4–84, median: 26). For the experiment, we chose the largest colonies, i.e., those containing minimum 24 workers, median: 32.5 (10 queenless; and 26 queenright colonies).

The nest sites used during this experiment were similar to the ones used during Experiment No. 1, but the sides of each nest site (both those ‘with a slit’ and those ‘without a slit’) were additionally covered with a small piece of transparent food foil; thus, these ‘slits’ were closed, preventing the air from flowing.

Experiment 3. If a hole in the red foil covering the nest site influences the nest site selection

On 3 July 2023, we collected 62 acorns with ant colonies: 27 queenless and 35 queenright (workers: 7–132, median: 27). For the experiment, we chose the largest colonies, i.e., those containing minimum 25 workers, median: 37.5 (13 queenless and 23 queenright colonies).

All the nest sites used in the experiment were similar to the sites ‘without slits’ used in Experiments No. 1 and No. 2.; however, one nest site was covered with a red filter, while the second was covered with a red filter with a hole. To achieve this, a small section was cut from all the pieces of red filter, and they were then stuck on using small pieces of adhesive tape; however, in the ‘experimental group’, a hole—approximately 10 mm × 1 mm—was left (see Figure 2b).

The ant colonies chosen for the experiment were assigned randomly to the Petri dishes. The position (i.e., left or right) of the nest sites was systematically varied to eliminate any chance of directional biases (see e.g., [25]). At the beginning of these experiments, each ant colony was placed into a separate Petri dish at a similar distance (approximately 7 cm) from the entrances to two artificial nest sites. The Petri dishes with ant colonies were kept in a thermostatic cabinet, where a daily cycle of 12 h: 12 h (light, 20 °C: dark, 10 °C) was maintained, which was the same as the artificial spring conditions previously used in experiments on *T. nylanderi* [26]. 

Each experiment lasted seven days. During this time, the ants were fed twice on dead *Drosophila hydei* and honey, and water was available ad libitum. The food was placed at a similar distance to the nest sites. Most ant colonies inhabited the nest sites during the first day; however, some of the colonies inhabited the sites at a later time, or they were at first split and later decided to inhabit a certain nest site. Thus, as the result of the experiments, we noted which nest cavity was inhabited by an ant colony after seven days. 

Chi-square tests were performed on the data obtained in the binary choice tests; colonies that split up between both nests (split colonies) and colonies that did not choose a nest (colonies out of nests) were not included in the analyses. All the statistical analyses were conducted using the software package Statistica, ver. 13 [27]. All the probability values shown are two-tailed.

## 3. Results

Experiment 1. If a ‘slit’ in the nest side influences the nest site selection

**Figure 3:**
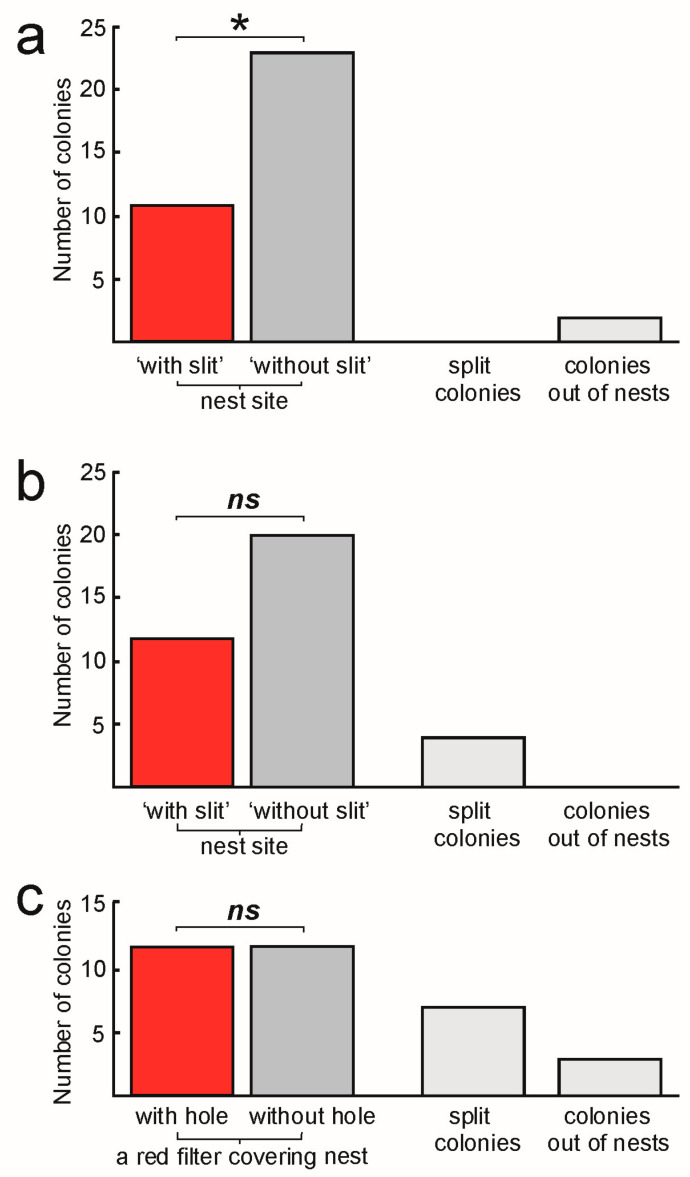
Outcomes of the binary tests on the nest site choice: the numbers of the cavity nesting ant *Temnothorax crassispinus* colonies which inhabited each nest type, or splitting into both nests, is shown. For each experiment, 36 colonies of the ants were used. (**a**) In Experiment 1, the ant colonies chose between nest sites ‘with a slit’ vs. those ‘without a slit’ (see Figure 1 and Figure 2 for the nest site design). (**b**) In Experiment 2, the colonies chose between nest sites ‘with a slit’ and ‘without a slit’; however, one side of all nest sites was additionally covered with a small piece of transparent food foil preventing air flow through the ‘slit’. (**c**) In Experiment 3, the nest sites were ‘without slits’, but in half of the sites, the red filter covering the nest sites contained holes (see Figure 1b). *—*p* < 0.05, *ns*—not significant, binomial test.

Seven days after the beginning of the binary choice test, 34 of the 36 colonies had chosen nest sites. The colonies preferred nest sites ‘without a slit’: 23 colonies inhabited sites ‘without a slit’, while 11 colonies sites ‘with a slit’ (*χ*^2^ = 4.24, *df* = 1, *p* = 0.040; analysis using the 34 colonies which inhabited nest sites; Figure 3a).

Experiment 2. If a ‘slit’ covered by food foil influences the nest site selection

After seven days, all the colonies inhabited nest sites; however, four of them split between the two available ones. There was no significant difference in the proportion of nest sites ‘with a slits’ and those ‘without slits’: 20 colonies chose sites ‘without a slit’, while 12 colonies chose sites ‘with a slit’ (*χ*^2^ = 2.00, *df* = 1, *p* = 0.16; analysis using the 32 colonies which chose one nest site; Figure 3b).

Experiment 3. If a hole in the red foil covering the nest site influences the nest site selection

After seven days, 33 of the 36 colonies inhabited nest sites; however, seven of them were split between the two available ones. An equal number of colonies inhabited nest sites with (13) and without a hole in the red foil covering the nest sites (13) (*χ*^2^ = 0.00, *df* = 1, *p* = 1.00; analysis using the 26 colonies which inhabited one nest site; Figure 3c).

## 4. Discussion

In this study, the cavity nesting ant *Temnothorax crassispinus* colonies more frequently inhabited nest sites ‘without a slit’ (Experiment 1). When such ‘slits’ in the nest sites were closed using transparent food foil (which prevented the air flow while keeping an inflow of light), there was no significant difference in the number of colonies inhabiting the two types of nest sites (Experiment 2). Additionally, the colonies inhabited the nest sites when half of these sites were covered with the red filter with a hole vs. those without hole in a completely random manner (Experiment 3). Splitting up between both nests (see results of the Experiments 2 and 3) could be explained by polydomy (i.e., simultaneous use of several nest sites by one colony [28]), which is a known behavior of many ant species, including the studied one [29]. During this study, we used queenright as well as queenless colonies. Nevertheless, we observed no differences in nest site selections by the two kinds of colonies. Additionally, as nest selection is based on the workers’ behavior, using such colonies did not affect the results of this study. The results of the experiments suggest that it is the air flow rather than the light inflow that affects the nest site selection. 

During several experiments, it has been shown that ants prefer dark nest sites (e.g., [12,14,30]). It was suggested that ants favor a dark interior, probably as an indirect cue of the nest wall’s integrity [14]. The result of the third experiment during this current study shows that a little additional light did not influence the nest site selection; however, the nest sites used in this study were made from acrylic plastic. By contrast with the natural cavities in wood or seeds, such artificial nest sites made with acrylic plastic (i.e., using a plexiglass frame) are not completely dark inside. The differences between ‘light’ and ‘dark’ sites used during other experiments were stronger; e.g., the nest sites used by Franks et al. [12,30] were constructed from pieces of cardboard sandwiched between microscope slides. During this study, the hole in the red foil covering the nest site added only a little light. Thus, the result of this study does not contradict the finding that ants favor a dark interior (cf. [14]). Nevertheless, all the nest sites used during this study were similar—i.e., using a plexiglass frame—and the additional light added through the hole in the red filter did not influence the nest site selection (see the results of Experiment 3). Additionally, during Experiment 2, more colonies (though not statistically significant) inhabited the nest sites ‘without slits’ even when the ‘slits’ were covered by transparent food foil. This could suggest that such a closed ‘slit’ is perceived by the ants. For ants, the sense of touch is important and can play a significant role with nest site selection. The most important part for sensory perception is antennae, and ants could perceive chemical, mechanical, and thermal stimuli using them [6]. For example, workers could be using their antennas to detect such a hollow and interpret it as a lower-quality potential nest site. However, using antennae, mechanical as well as chemical stimuli (e.g., odors and flavors, water, CO_2_) can be perceived by ants [6], and we believe that air flow rather than just touch was used as the ants during the nest site selection. Another limitation of the study is the *p* value for the results of Experiment 1—the *p* value (0.040) is not very far from the level of significance (*p* = 0.05) typically used in biology. Thus, it would be good to repeat this experiment preferably using another species.

Under natural conditions, cavity nesting ant colonies frequently inhabit old, empty acorns. Such acorns are brittle and could have cracks [22], which in this study were mimicked by ‘slits’ made in the artificial nest sites. The results of the study show that artificial nest sites ‘without slits’ are preferred by the cavity nesting ants, and probably the air flow is perceived by workers to assess the quality the nest site. Cracks in the nest sites inhabited by cavity nesting ants could affect the temperature, as well as the humidity, inside such a nest site and therefore the brood development. However, what is probably more important for ant colonies is that such damage reduces the strength and has an effect on the durability of such a nesting site. Cavity nesting ants are able to improve their nest sites, e.g., they decrease the entrance size using different materials [11,18,20,31]. Thus, cracks in walls of acorns could potentially also be repaired using different materials. Indeed, during this study, we observed that some colonies put some loose material onto the ‘slits’, as well as onto the entrances; however, during this study, not many materials (i.e., a few parts of acorns) were available in the Petri dishes for such modifications. Nevertheless, such repairs could probably prevent the air inflow, but they would not strengthen the acorn wall considerably. Additionally, as the acorn’s wall is thin, the acorns used as nest sites ensure protection for the ant colonies from potential enemies rather than from physical factors like extreme temperatures. Therefore, cracks could provide additional entrances for pests and brood parasites, and the presence of such structures could reduce the ants’ ability to defend the nests. Thus, the possibility to easy recognize if a potential nest site is strong, i.e., without cracks, could be favorable.

## Data Availability

The data presented in this study are available on request from the correspondence author.
